# Assessing the Awareness and Understanding of Hospital Triage Among the General Population of Al-Ahsa, Saudi Arabia

**DOI:** 10.7759/cureus.53864

**Published:** 2024-02-08

**Authors:** Amjad A AlNaim, Noura A AlNaim, Ayah F Albash, Maryam A Almulhim, Latifah A Albash, Nasser Almulhim

**Affiliations:** 1 Medicine and Surgery, King Faisal University, Hofuf, SAU; 2 Emergency Medicine, King Fahad Hospital, Hofuf, SAU

**Keywords:** awareness, emergency department, saudi arabia, emergency medicine, triage

## Abstract

Background

Hospital triage is a critical process in emergency departments (EDs) worldwide. The efficiency of the triage process significantly impacts the overall functioning of the ED and patient outcomes. However, the effectiveness of triage is not solely dependent on the healthcare professionals conducting it. The awareness and understanding of the triage process among the general population also play a crucial role.

Methods

This study aimed to assess the awareness and understanding of hospital triage among the general population of Al-Ahsa. A cross-sectional design was conducted in Al-Ahsa, Saudi Arabia, from July to September 2023. Data were collected using an online questionnaire.

Results

This study examined the awareness, understanding, attitude, and socio-demographic factors of hospital triage among 389 participants in Al-Ahsa, Saudi Arabia. Results showed that 59.4% (n=231) of participants were aware of emergency triage, with 91.8% (n=457) agreeing with patient classification based on deterioration. Expectations for waiting time varied, with 38.8% (n=151) expecting 5-10 minutes. Participants expressed positive attitudes, with 91% (n=354) believing triage improves patient care. Socio-demographic analysis revealed higher awareness among younger age groups, males, and highly educated individuals. Educational level was associated with participants' attitudes. These findings emphasize the importance of targeted awareness campaigns and improved waiting room amenities for effective hospital triage.

Conclusion

The study found that public awareness of emergency triage is average, with high satisfaction with the concept of patient classification based on deterioration. Periodic health education sessions regarding the importance of ER triage are recommended for healthcare visitors and staff.

## Introduction

Emergencies are high-risk circumstances in which a person's bodily or mental health is unexpectedly compromised, necessitating immediate, significant action [1,2]. According to World Health Organization data, trauma and accident patients occupy one-third of hospital beds. First taken to hospital emergency departments, these people cost the world more than $500 million as a result [3-5]. Triage is a French term that refers to grouping patients based on their circumstances and individual needs [5-7]. For the triage of emergency patients, numerous approaches have been developed with varying advantages and disadvantages. The Emergency Severity Index (ESI), a five-tier triage method, is praised for its simplicity, ease of training, perceptual approach, and capacity to operate in most EDs worldwide [8]. When patients arrive at the ED, triage is an important step. The management of patients in the ED depends on this task, which is extremely laborious and difficult to do when the ED is busy [8-12].

Patients are at level 1 triage if they require a life-saving procedure. The patient is categorized as level 2 if they have a reduced degree of consciousness, excruciating pain, or excruciating distress. In clinical settings, patients are classified as level 3 when they necessitate multiple diagnostic procedures (including hematological or urinalysis, electrocardiograms, radiographic examinations, etc.) yet exhibit no significant alterations in vital signs. Level 4 classification is applied to those requiring only a singular diagnostic facility. Furthermore, level 5 is assigned to patients who do not demand any supplementary services. The primary objective of triage is to promptly identify individuals presenting with acute or potentially fatal conditions, initiate requisite interventions, and subsequently guide them to the appropriate sector within the emergency department [13-15]. It is imperative to accord immediate medical attention to those encountering critical conditions such as cardiac arrest, airway blockages, or shock, with the aim of preserving life.

Nevertheless, overcrowding within emergency departments (EDs) can detrimentally impact the standard of care by necessitating the dispersion of resources among patients in need of urgent medical attention and those with less pressing health concerns [10,16]. Crowding in EDs has been consistently recognized as a crucial factor affecting patient satisfaction [17]. In economically disadvantaged nations, unlike their affluent counterparts, emergency healthcare services, including triage, often represent a vulnerable component of the healthcare system. However, with effective organization, these services can yield significant life-saving and cost-efficient outcomes [18]. A notable number of hospitals in developing countries lack a formalized triage system [19]. In such environments, emergency care (EC) is typically nonexistent, and patients are treated in wards or outpatient clinics based on their point of entry. Healthcare professionals frequently adopt a "first come, first served" approach in patient consultation [20]. This practice can lead to perilous delays for critically ill patients [21]. Furthermore, once a patient is identified as critically ill, additional delays in commencing emergency treatment may occur [18].

Unfortunately, there are no clear standard national nurse training programs for triage yet. If nurses employ standard procedures and have a thorough understanding of triage, the benefits of this approach will be evident in the interventions they do for patients. Hospitals in Iran, which serve as the standard for establishing the triage system, have not received any information on a national triage scale [17].

The awareness and understanding of hospital triage among the general population is an important factor that influences the utilization and perception of ED services. A high level of awareness and understanding can enhance patient compliance, cooperation, and trust in the triage system, as well as reduce unnecessary visits and complaints. Conversely, a low level of awareness and understanding can lead to frustration, confusion, and dissatisfaction among patients and their families, as well as increased workload and stress for ED staff.

Considering the significance of raising public awareness and comprehension of hospital triage, there is a lack of studies on this topic in Saudi Arabia, particularly in Al-Ahsa, one of the biggest governorates in the Eastern province. There are several health issues in Al-Ahsa, a large geographical area with a population of roughly 1.3 million people, including a high incidence of chronic diseases, traffic accidents, and infectious diseases [22,23].

Therefore, this study aims to assess the level of awareness and understanding of hospital triage among the general population of Al-Ahsa, Saudi Arabia, and to identify the factors associated with it. The findings of this study can provide valuable insights for health policymakers, managers, and practitioners to improve the triage system and public education on it.

## Materials and methods

Study settings

This cross-sectional study was conducted from 2023 to 2024 by using a convenient sampling technique in Al-Ahsa, Eastern region of Saudi Arabia, to assess the level of awareness and understanding of hospital triage among the general population of Al-Ahsa and to identify the factors associated with it. The methods that have been used in this study were reported in line with Strengthening the Reporting of Observational Studies in Epidemiology (STROBE) guidelines.

Inclusion and exclusion criteria

An online questionnaire was distributed via social media. In context to the study objectives, an Arabic survey was used to suit the general public, which has been priorly used and ensured to have acceptable validity and reliability. The included criteria were as follows: people who are living in the Al-Ahsa, Eastern region in Saudi Arabia, between 18 years old and older. The exclusion criteria were as follows: people from areas other than Al-Ahsa who are younger than 18 years of age. In addition to subjects who did not fulfill eligibility criteria, all the participants who refused to give their consent or reported missing data were excluded. Diversity in distributing the survey on different social media applications and at multiple different times was applied to reduce the selection sampling bias.

Assessment tool

The survey was divided into three main sections: (A) four questions about participation consent, naturality, age of more than 17 years, and residence to ensure eligibility of all subjects, (B) three questions regarding socio-demographic data, which included age, gender, and education level, (C) nine questions to explore awareness and understanding of hospital triage. The purpose of the study and the estimated time to answer the questions were provided with online consent, which was obtained before filling out the survey.

Sample size and statistical analysis

The sample size was determined to be 385 subjects using the Richard Geiger equation, with a margin error of 5%, a confidence level of 95%, a population of 1,104,267, and 50% for response distribution. SPSS, version 26.0 (IMB, Inc., Armonk, US), was used to analyze the data, and Microsoft Excel 2016 (Microsoft® Corp., Redmond, US) was used to present the data in tables and graphs. The frequency of the questions was calculated to show the total responses for each option in the survey. Most of the questions were qualitative, and the chi-square test was used as the main statistical analytical test to assess relationships between different study variables. A p-value of less than 0.05 was deemed statistically significant.

Ethical considerations

The consent for voluntary participation was obtained from all participants after declaring the study objectives and expected filling time in the first section of the questionnaire. Respondent anonymity and confidentiality were guaranteed. The research complies with the ethical standards and is approved by the ethical committee of the Deanship of Scientific Research at King Faisal University, KFU-REC-2023-OCT-ETHICS1294.

## Results

A total of 389 eligible participants from Al-Ahsa filled in the study questionnaire. Participants' ages ranged from 18 to 80 years, with a mean age of 35.1±11.7 years. A total of 298 (76.6%) respondents were females. According to educational level, 105 (27%) had a secondary level of education/diploma or below, 258 (66.3%) had a bachelor's degree, and only 26 (6.7%) had a post-graduate degree (see Table 1).

**Table 1:** Demographic characteristics of the study population

Demographic data	n	%
Age in years		
18-35	256	65.8
36-50	68	17.5
51-80	65	16.7
Gender		
Male	91	23.4
Female	298	76.6
Educational level		
Secondary and below	105	27.0
Bachelor degree	258	66.3
Post-graduate	26	6.7

A total of 231 (59.4%) participants heard about the concept of emergency triage, 142 (36.5%) think all patients need to be visited by a doctor within the first five minutes, and most of them (91.8%) agree with the classification of patients based on the deterioration. One hundred and fifty-one (38.8%) expect the reasonable duration of waiting when visiting a doctor should be within five to 10 minutes, 140 (36%) expect it should be within 10 to 20 minutes, and only 42 (10.8%) expect it should be less than five minutes.

**Table 2:** Awareness and understanding of hospital triage among the general population of Al-Ahsa, Saudi Arabia

Awareness	n	%
Have you heard about the concept of emergency triage?		
Yes	231	59.4
No	158	40.6
Do you think all patients need to be visited by a doctor within the first five minutes?		
Yes	142	36.5
No	247	63.5
Do you agree with the classification of patients based on the deterioration?		
Yes	357	91.8
No	32	8.2
What is your expectation of the reasonable duration of waiting when visiting a doctor?		
Less than five minutes	42	10.8
5-10 minutes	151	38.8
10-20 minutes	140	36.0
20-30 minutes	56	14.4

**Figure 1:**
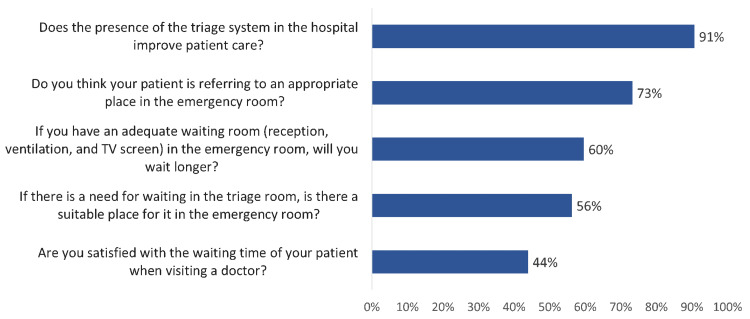
Public attitude and satisfaction towards hospital triage concept in Al-Ahsa, Saudi Arabia

The majority (91%) of the respondents think that the presence of the triage system in the hospital improves patient care, 73% think that patients are referred to an appropriate place in the emergency room, and 60% reported they would wait longer if there would be an adequate waiting room (reception, ventilation, and TV screen) in the emergency room, 56% were satisfied with the waiting place in the emergency room, and 44% were satisfied with the waiting time of patient when visiting a doctor (Figure 1)

Exactly 69.1% of participants aged 18-35 were aware of emergency triage compared to 36.9% of others aged 51-80 with recorded statistical significance (p=0.001). Also, 70.3% of male participants were aware of emergency triage versus 56% of females (p=0.015), and 88.5% of highly educated participants were aware of emergency triage in comparison to 61% of others with a lower educational level (p=0.005; Table 3).

**Table 3:** Relation between public awareness of emergency triage and their socio-demographic data ^ Exact probability test * p<0.05 (significant)

Factors	Have you heard about the concept of emergency triage?				p-value
	Yes		No		
	n	%	n	%	
Age in years					0.001*
18-35	177	69.1	79	30.9	
36-50	30	44.1	38	55.9	
51-80	24	36.9	41	63.1	
Gender					0.015*
Male	64	70.3	27	29.7	
Female	167	56.0	131	44.0	
Educational level					0.005*^
Secondary and below	64	61.0	41	39.0	
Bachelor degree	144	55.8	114	44.2%	
Post-graduate	23	88.5	3	11.5	

Only educational level was associated with the study participants' attitudes, as all participants with high education agreed that the triage system in the hospital improves patient care versus 88.6% of others with lower levels of education (p=0.048; Table 4).

**Table 4:** Relation between public attitude and perception of emergency triage and their socio-demographic data ^ Exact probability test * p<0.05 (significant)

Factors	The presence of the triage system in the hospital improves patient care				p-value
	Yes		No		
	n	%	n	%	
Age in years					0.259
18-35	231	90.2	25	9.8	
36-50	65	95.6	3	4.4	
51-80	57	87.7	8	12.3	
Gender					0.862
Male	83	91.2	8	8.8	
Female	270	90.6	28	9.4	
Educational level					0.048*^
Secondary and below	93	88.6	12	11.4	
Bachelor degree	234	90.7	24	9.3	
Post-graduate	26	100.0	0	0.0	

## Discussion

The present study was conducted to evaluate the general public's awareness and comprehension of hospital triage. The emergency department is a critical component of the healthcare system [24]. ED staff serve as the initial contact for individuals facing severe, life-threatening conditions [25]. The caliber of care in the ED significantly influences patient survival, their care experience and satisfaction, and their psychological health [26]. Efficient operation of the ED can enhance patient throughput in various hospital departments, particularly for those in need of urgent medical interventions [27].

The study revealed that over half of the participants were familiar with the concept of emergency triage, while approximately a third believed that every patient should be seen by a doctor within the first five minutes. A majority concurred with patient categorization based on their health deterioration. Additionally, over a third of respondents expected a reasonable waiting time to see a doctor to be between five to 10 minutes, with a similar percentage anticipating a 10 to 20-minute wait, whereas only a tenth expected it to be under five minutes. Alsulimani et al. [28] found comparable results, with 52% of Saudi participants having an accurate understanding of triage. The study also noted that 80% understood the reason why some patients received quicker access to a room, and 85.3% deemed this prioritization fair. Seibert et al. [29] reported that 68% of participants had an understanding of this concept, and a higher understanding was correlated to increased satisfaction regarding its fairness. Almadhyan et al. [30] observed that 54.6% were aware of the triage system in EDs, but 63.4% were unaware of urgent care centers providing services outside of EDs, and 47.4% did not realize that health or urgent care centers could handle most emergencies. These findings contrast with those of Alhabdan et al. [31], who reported that only 11% understand hospital triage in their investigated sample. Moreover, reports of studies conducted in the UK and Australia demonstrated hospital triage understanding by 33% and 50%, respectively [32-33]. Meek et al. [34] recorded a score of six out of seven for perceived fairness in triage. Furthermore, 24% of respondents understood the triage process, but only 45% had a correct understanding, implying that merely 11% of participants could accurately define triage.

Regarding public satisfaction and attitude towards ED triage, the current study showed that most of the participants think that the presence of the triage system in the hospital improves patient care, about three-fourths think that patients are referred to an appropriate place in the emergency room, and two-thirds reported they would wait longer if there would be an adequate waiting room (reception, ventilation, and TV screen) in the emergency room. Also, more than half were satisfied with the waiting place in the emergency room, and less than half of the respondents were satisfied with the waiting time of patients when visiting a doctor. Özhanlı et al. [35] found that the mean score for the Triage Satisfaction Scale was 7.37±2.11, while the mean score for the Newcastle Satisfaction with Nursing Scale was 73.34±17.6 indicating a high satisfaction rate. According to multiple reports, including studies conducted by Şahbaz-Karagün in the ED of the university hospital, Aşılıoğlu et al. in the pediatric emergency department, and Şanlı et al., the general satisfaction rates were found to be 64.2%, 74.6%, 97%, respectively [36-38].

This investigation acknowledges several intrinsic limitations. Primarily, the dependency on convenience sampling via social media platforms may induce a sampling bias, potentially not representing the broader demographic of Al-Ahsa, Saudi Arabia. Employing an online survey method could restrict participation from those with limited internet access or digital literacy. The inherent nature of self-reporting in the survey might lead to response bias, with participants possibly offering socially favorable responses over their genuine beliefs or experiences. Additionally, the study's concentration on a singular geographic locale could impede the extrapolation of its results to different environments or demographic groups. Lastly, the cross-sectional framework of the research offers a temporal overview of awareness and comprehension yet lacks the capability to monitor temporal variations or establish causal connections.

## Conclusions

In conclusion, the study revealed that public awareness and understanding regarding emergency triage was average (intermediate level), mainly indicating the importance of classification of patients based on their deterioration. Also, the public attitude towards ED triage was high, with reported high satisfaction with the concept, mainly concerning ED, waiting area, and physician care. Periodic health education sessions are needed for healthcare visitors and staff regarding the importance of the emergency room triage concept and the importance of their attitude toward the patient's preferences regarding the timing of the care provided.
